# WWJD?

**DOI:** 10.4269/ajtmh.18-0702

**Published:** 2019-01

**Authors:** Jason M. Nagata

**Affiliations:** Division of Adolescent and Young Adult Medicine, Department of Pediatrics, University of California, San Francisco, San Francisco, California

I first encountered global health through the writings of Dr. Paul Farmer on his work in Haiti. Farmer argued that inequalities led to infections and criticized “Pathologies of Power,” linking poverty to the causation of disease. In college, I read all things Paul Farmer and would often ask myself, “WWPD?” (What would Paul do?) if he were in my situation. I dreamed that I would study medicine and anthropology and find a place, like Haiti for Paul Farmer, where I could start a clinic in an underserved area, conduct social medicine research, and return at every possible opportunity during my training.

I won a yearlong fellowship to study medical anthropology at Oxford and gained admission to medical school at the University of California, San Francisco. I was thrilled. I was following in the footsteps of Farmer. I planned to research food insecurity and the human immunodeficiency virus (HIV) in rural Kenya. My Asian tiger parents, although thrilled that I would be a doctor with pedigree, were horrified that I would be living in a “third world” country. My dad e-mailed me daily U.S. State Department updates about violence, terrorism threats, and disease outbreaks in Kenya before my departure. A week before I was set to leave for Kenya, he discovered emerging cholera outbreaks around Lake Victoria, where I would be staying. Alarmed, my parents flew from Los Angeles to London before my departure, bringing an entire suitcase full of water treatment methods to ensure I did not die from cholera and diarrhea. We practiced each step of the handheld water filtration pump in detail every day. We reviewed protocols for requesting air evacuation from my insurance plan in case of emergencies.

The journey from Oxford to Mfangano Island, Kenya, took 2 days. After a 7-hour flight to Nairobi, I made a connecting flight overlooking the savannah plains of the Maasai Mara National Reserve on a loud rickety propeller plane, and then took a bumpy bus ride on roads of cracked pavement followed by a glide on a worn wooden fishing boat, arriving to the island at dusk. I rode on the back seat of a motor bike grasping my water treatment–filled suitcase for dear life, feeling insects accrue on my face as we whisked through swarms of buzzing mosquitoes.

The island lacked electricity, Internet, telephone lines, paved roads, and running water. Each morning, I accompanied my host mother, Akeyo, on the 2-mile-long journey through tortuous footpaths to the shore of Lake Victoria to gather our daily water. Whereas I only needed to carry the plastic jug that I would drink from throughout the day, Akeyo balanced a massive vat on her head that provided sustenance for her entire family—including eight children—and all of the cooked meals for the day. Like Wonder Woman, she traversed the return journey with a neck of steel in a sapphire sarong and sandals worn down to the sole. After we returned to our thatched roof, mud-walled home, Akeyo boiled the water over a wood fire. When my ration of water was boiled, I let it cool and then started my hand-pumped filtration system. To the filtered water, I dissolved one chlorine tablet, followed by two drops of iodine, as if I were in a chemistry laboratory. All in all, the water cleaning process took 3 hours each day.

Akeyo chuckled with a singsong laugh as she watched my daily ritual, but also encouraged me to use all of the techniques because she did not want me to get sick under her watch. Otieno, my sinewy host father, returned home for lunch from farm labor as I finished my water ritual. Beads of perspiration trickled down his skin, wrinkled from decades of the sun’s abuse. With his calloused hands, he pumped the water filtration contraption a few additional times. I offered to treat my host family’s water as well.

Otieno declined, saying, “Our stomachs are accustomed to the lake water.” He grinned, revealing his bright white teeth, adding, “Your stomach is weak!” It felt wrong that the water I drank was more disinfected than the water my host family consumed. But I also realized that the period when I could offer my filter and disinfectants represented only a short blip in their lifetime exposure to water-borne diseases. To my host parents’ credit (and to my biological parents’ relief), I never once had any gastrointestinal problems during my 2-month stay in Kenya.

I realized that with all of the time I spent guarding against possible water-borne tropical diseases, I only had a couple of hours each afternoon to actually conduct my research interviews. From these interviews, I learned that virtually all people with HIV on the island experienced food insecurity and worried about how they would feed their families. Without access to basic requirements for survival such as food and water, it is difficult to ascend the ladder of Maslow’s hierarchy to other domains of life.

I returned to Oxford in time for the formal dinner celebrating the beginning of the spring Trinity Term. Young men donned tuxedos with long black robes, called “sub-fusc,” whereas young women revealed their tans from exotic destinations under low-cut gowns. The Hogwarts-style dining hall bustled with excitement. I talked about the water situation in Lake Victoria, the doctoral student seated on my left discussed Palestinian geopolitics, and the fellow seated on my right critiqued neocolonialism in Myanmar. Amid the chatter, nobody finished the servings of their four-course meals. We were offered four types of sparkling water; my neighbor across the table returned her Perrier because she felt it was too flat. I excused myself from dinner, feeling sick with indigestion through the transition from eating with my hands with Akeyo and Otieno to the crème brûlée dessert at a formal hall. I went to the loo, threw up in the marble toilet, and had diarrhea for the first time in 2 months.

Transitions between different contexts, time zones, and cultures were more difficult than I had anticipated. During his preclinical years of medical school, Paul traveled from Harvard to Haiti so often that he acquired the nickname “Paul Foreigner,” while still managing to excel academically. When I started medical school the following year, I initially dreamed that I too would be jet-setting across the globe every long weekend. However, I soon gave up on that romantic notion. Medical school was exhausting, and catching up on sleep debt during breaks was more essential than accruing more financial debt from international travel, dealing with layovers and jetlag, and having to worry about clean water.

Although I realized that the Paul Farmer lifestyle could not be sustainable for me, I still wanted to find a way to engage with global health throughout my medical training. I began to relate more to his partner in crime, Dr. Jim Kim. Jim had practical Asian immigrant parents, similar to mine. When Jim had decided after his first semester of college that he would be studying politics and philosophy, his father said, “After you finish your medical residency, you can do whatever you want.” Jim’s career took the more pragmatic approach, and he was able to effectively advocate for global health issues through leadership with the World Health Organization, Dartmouth College, and the World Bank. I began to ask myself: “WWJD?” (What would Jim do?). I even crashed Dartmouth College alumni events to have the opportunity to meet Jim, even though I had no affiliation with the university.

I began working on systematic reviews with the World Health Organization that informed HIV guidelines from the carrels of my university library. I also conducted secondary analyses of Kenyan data, evaluating nutrition interventions for people living with HIV and trying to understand the complex relationships between undernutrition and HIV, work that built on my master’s fieldwork but that did not require frequent travel. Given advances in modern technology and social media, I could easily schedule conference calls with Kenyan research colleagues and keep up with my host family’s updates on Facebook. Although I sometimes felt like a “limousine liberal,” doing global health research from the comfort of my home, it was the only way that I could engage globally while balancing the grueling clinical demands of medical training.

I am writing this essay on a flight to Kenya, my first visit after an 8-year hiatus. The faces and stories of Akeyo, Otieno, and the interview participants I encountered years ago remain fresh in my memory. As I complete my training and launch my professional career this year, I wonder to myself, “WWJD?”

